# A report of two infant cases operated for jejunal duplication cyst associated with malrotation and volvulus

**DOI:** 10.1016/j.ijscr.2020.02.009

**Published:** 2020-02-07

**Authors:** Ahmed Azzam, Ali N. Abdulkarim, Ahmed E.M. Shehata, Ibrahim Mahran, Ahmed Arafa, Ahmed Arafat, Sherifa Tawfik, Muayad Shaban, Aliyu Anache, Sherif Kaddah, Heba Taher

**Affiliations:** a Pediatric Surgery Department, Cairo University, Egypt; b Ministry of Health and Population, Egypt; c Umar Yar‘Adua Maternal and Children Hospital Katsina, Nigeria; d Faculty of Medicine, Cairo University, Cairo, Egypt

**Keywords:** Intestinal duplication, Intestinal malrotation, Intestinal obstruction, Bilious vomiting

## Abstract

**Introduction:**

Both Enteric duplication and intestinal malrotation are concerning causes for intestinal obstruction in the pediatric age group and they very rarely coexist in the same patient.

**Presentation of cases:**

We present 2 cases of previously healthy children, the first is a 4-month-old infant and the second is a 1.5-year-old boy, both presented with recurrent attacks of bilious vomiting that proved to be due to acute midgut volvulus caused by an enteric duplication cyst associated with intestinal malrotation.

**Discussion:**

Enteric duplication and intestinal malrotation are two of the concerning causes of billious vomiting in the pediatric age group. They could be encountered at any level of the alimentary tract from the tongue to the anus. The term malrotation refers to all abnormalities of intestinal position. The coincidence of intestinal malrotation and an enteric duplication cyst (EDC) is very rare and has been described only in a few case reports.

**Conclusion:**

The concomitance of EDC and intestinal malrotation is extremely rare and should be kept in mind in a child presenting with bilious vomiting especially in a child preoperatively diagnosed with a duplication cyst.

## Highlights

• THE COMBINATION OF Enteric duplication and intestinal malrotation IS rare but can coexist.
• Malrotation MUST BE kept in mind in patients with preoperatively diagnosed duplication cyst.
• Surgical correction in form of A LADD's procedure and resection of THE duplication WITH anastomosis IS CURATIVE.
• Ultrasound seems TO BE a good diagnostic tool for THE PREOPERATIVE diagnosis of both conditions.

## 1 Introduction

Enteric duplication is defined as a cystic or tubular structure that contains a well developed smooth muscular wall and is lined by a mucous membrane whereas intestinal malrotation refers to any change of the normal intestinal position and attachment [1,2]. Both Enteric duplication and intestinal malrotation are concerning causes for intestinal obstruction in the pediatric age group and they very rarely coexist in the same patient [3,4]. Complications of enteric duplications include acute distention or infection of the cyst, ulceration, bleeding and rarely perforation [1,5]. Management of enteric duplication entails excision of the cyst along with the adjacent bowel segment with primary anastomosis usually via laparotomy [5].

Here we present 2 cases of previously healthy children, the first is a 4-month-old infant and the second is a 1.5-year-old boy, both presented with recurrent attacks of bilious vomiting that proved to be due to acute midgut volvulus caused by an enteric duplication cyst associated with intestinal malrotation.

## 2 Cases presentation

### 2.1 Case 1

A 4-month-old infant presented to our emergency department with a history of repeated attacks of bilious vomiting that started 2 weeks after birth. His examination revealed lax abdomen and large abdominal cystic lesion in the right hypochondrium.

**Fig. 1:**
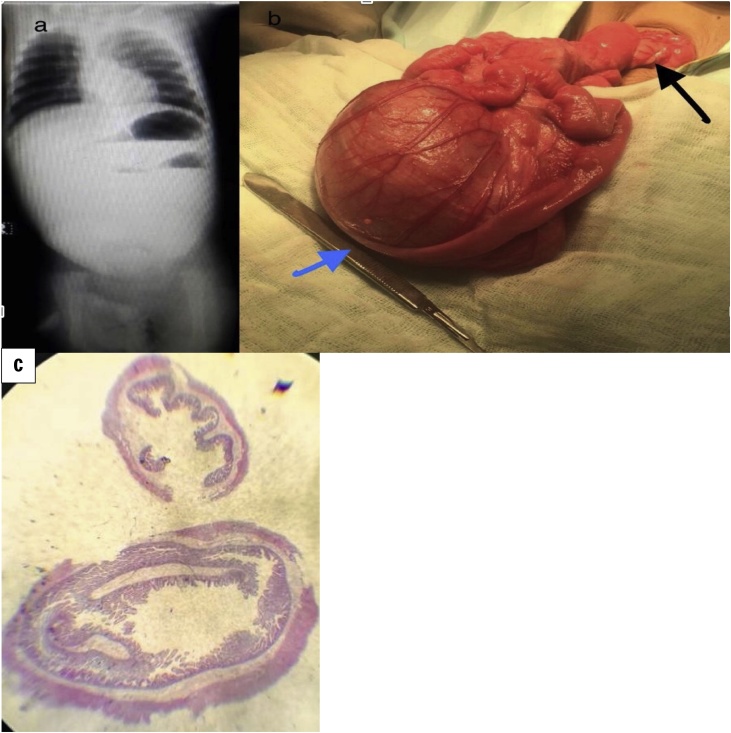
a: Abdominal X-ray showing multiple fluid levels and Bowle obstruction, b: black arrow shows twist in the mesentry and blue arrow jeujenal duplication cyst, c:microscopic picture of jeujenal duplication.

Radiological assessment entailed an erect abdominal X-ray which showed 3 gas bubbles and distal gasless abdomen (Fig. 1a) and an abdominal ultrasound which was notable for a large cyst measuring 56 × 73 × 86 mm and abnormal superior mesenteric vessels orientation and characteristic whirlpool sign.

After initial resuscitation, abdominal exploration was done which showed malrotation, midgut volvulus, and jejunal duplication cyst (Fig. 1b). Ladd’s procedure was done along with excision of the affected jejunal segment, jejune-jejunal anastomosis and prophylactic appendectomy. Oral feeding was commenced on the third day postoperatively and the patient was discharged on the sixth day with no postoperative complications.

Histology (Fig. 1c) of the resected specimen revealed an edematous intestinal wall with outer surface granulation tissue. Attached developmental cyst with inflamed muscular wall and granulation tissue, confirming the clinical picture of duplication cyst.

### 2.2 Case 2

A 1.5-year-old male presented to our emergency department with a history of recurrent attacks of bilious vomiting that started since he was 6 months old. Abdominal examination revealed a palpable huge cystic mass and bloody stool per PR. Abdominal ultrasound revealed evidence of markedly dilated small bowel loops with an abnormal rotation of the bowel and the umbilical region.

**Fig. 2:**
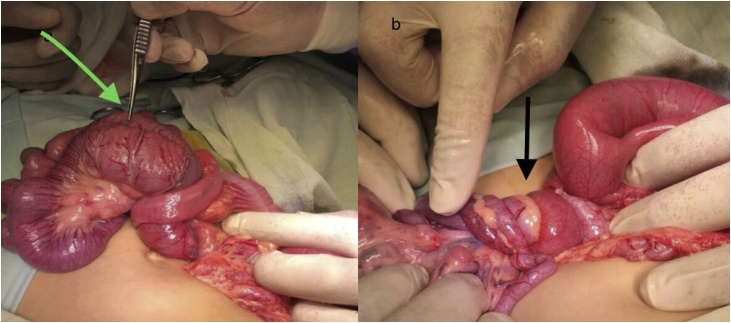
a: Green arrow jeujenal duplication, b: black arrow twist in the mesentry.

After adequate preoperative preparation, abdominal exploration via transverse supraumbilical incision was performed. Ladd’s procedure was performed and excision of the duplicated jejunal segment (10 cm long) 100 cm from duodenojejunal flexure (Fig. 2a, b). The jejune-jejunal anastomosis was performed using along with prophylactic appendectomy. The postoperative course was uneventful.

Patient started oral intake after 3 days.

Histopathological examination of the excised segment revealed a portion of jujenal wall with underlying cyst formed of intestinal wall, with preserved muscle layer and regularly scattered neural elements, with small intestinal mucosal lining. In addition, there were areas of devitalization with moderate congestion and edema. Margins were free and viable

## 3 Discussion

Enteric duplication and intestinal malrotation are two of the concerning causes of bilious vomiting in the pediatric age group. Enteric duplication is defined as a cystic or tubular structure that contains a well developed smooth muscle layer and lined with gastrointestinal mucosal lining. They could be encountered at any level of the alimentary tract from the tongue to the anus [1]. The term malrotation refers to all abnormalities of intestinal position and attachment [2]. The excessive length of the mesentery or a point of adhesion at the convexity of the loop can act as an axis for a twist of the midgut causing acute midgut volvulus [2]. The coincidence of intestinal malrotation and an enteric duplication cyst (EDC) is very rare and has been described only in a few case reports [3,4].

75% of patients with duplication cysts present within the first year of life usually with a picture of abdominal pain, vomiting or an abdominal mass. However, it may remain asymptomatic and accidentally discovered during laparotomy [1,3,6]. Acute distention or infection of the retained secretions within the cyst can cause a picture of acute abdomen. Other complications include ulceration, bleeding and rarely perforation as in 17 %–36 % of the cases the cyst contains heterotropic gastric mucosa [1,5]. EDC can cause intestinal obstruction by acting as a leading point for intussusception or by a huge duplication cyst causing compression on the adjacent bowel [5].

Diagnosis of EDC can be reached through abdominal ultrasound preoperatively which can reveal an anechoic mass in the absence of ulceration and bleeding. The cyst’s wall is typically 2−3 mm in thickness and the mucosa shows a characteristic echogenic signal inside the cyst. Ultrasonography can also diagnose prenatal EDC [5,7].

Other diagnostic modalities like plain X-ray, Computerized tomography (CT), Magnetic Resonance Imaging (MRI) can aid in the diagnosis and the localization of the duplication cyst.

technetium-99 m pertechnetate scan can be helpful in diagnosing Duplication cysts containing gastric mucosa and may differentiate them from mesenteric cysts [5,8].

Management of cystic EDC entails laparotomy with resection of the cyst and the adjacent bowel segment with primary anastomosis [5]. As both our patients had concomitant acute midgut volvulus, urgent exploration and Ladd’s operation were performed along with prophylactic appendectomy to avoid diagnostic dilemmas in the future.

## 4 Conclusion

The concomitance of EDC and intestinal malrotation is extremely rare and should be kept in mind in a child presenting with bilious vomiting especially in a child preoperatively diagnosed with a duplication cyst. Preoperative ultrasound seems a reliable preoperative diagnostic modality.

The work has been reported in line with the SCARE criteria [9].

## Declaration of Competing Interest

We declare no conflict of interest.

## Sources of funding

This research did not receive any specific grant from funding agencies in the public, commercial, or not-for-profit sectors.

## Ethical approval

Our study is exempted from ethical approval in Institutional Review Board of our hospital.

## Consent

We have written informed parental consent for the two patients involved in this report for publication.

## Authors contribution

Ahamed Azzam: operated on both cases and collected pictures.

Heba Taher: Consultant in charge of case 1 operated supervised the writing process and corresponding author.

Ahmed Arafa: consultant in charge case 2.

Ali Nabil abdulkarim: literature search and drafting manuscript.

Ahmed E.M. Shehata: literature search and drafting the manuscript.

Ibrahim Mahran: operated on and assisted in case one and helped with drafting.

Sherif Kaddah: head of Pediatric surgical department encouraged writing the manuscript.

Aliyu Ahmed Anche: pediatric surgeon Turai Umar Yar‘adua maternal and children hospital Nigeria preparation of presentation.

Sherifa tawfik: pathologist.

Muayad shaban: preparation of pathology slides slides.

## Registration of research studies

This is a case report and not a human study. It is exempt from registering.

## Guarantor

Heba Taher.

## Provenance and peer review

Not commissioned, externally peer-reviewed.
